# Analysis of clinical phenotypic and genotypic spectra in 36 children patients with Epilepsy of Infancy with Migrating Focal Seizures

**DOI:** 10.1038/s41598-022-13974-9

**Published:** 2022-06-17

**Authors:** Haiyan Yang, Xiaofan Yang, Fang Cai, Siyi Gan, Sai Yang, Liwen Wu

**Affiliations:** 1 Department of Neurology, Hunan Children’s Hospital, Ziyuan Road 86th, Changsha, 410007 Hunan People’s Republic of China; 2 Department of Pediatrics, Qilu Hospital of Shangdong University, Jinan, People’s Republic of China; 3 Department of Neurology, Chenzhou No 1 People’s Hospital, Chenzhou, People’s Republic of China

**Keywords:** Genetics, Neuroscience

## Abstract

Epilepsy of Infancy with Migrating Focal Seizures (EIMFS) is a rare developmental and epileptic encephalopathy (DEEs) with unknown etiology, and poor prognosis. In order to explore new genetic etiology of EIMFS and new precision medicine treatment strategies, 36 children with EIMFS were enrolled in this study. 17/36 cases had causative variants across 11 genes, including 6 novel EIMFS genes: *PCDH19*, *ALDH7A1*, *DOCK6*, *PRRT2*, *ALG1* and *ATP7A*. 13/36 patients had ineffective seizure control, 14/36 patients had severe retardation and 6/36 patients died. Of them, the genes for ineffective seizure control, severe retardation or death include *KCNT1*, *SCN2A*, *SCN1A*, *ALG1*, *ATP7A* and *WWOX*. 17 patients had abnormal MRI, of which 8 had ineffective seizure control, 7 had severe retardation and 4 died. 13 patients had hypsarrhythmia, of which 6 had ineffective seizure control, 6 had severe retardation and 2 died. Also, 7 patients had burst suppression, of which 1 had ineffective seizure control, 3 had severe retardation and 3 died. This study is the first to report that *ALDH7A1*, *ATP7A*, *DOCK6*, *PRRT2*, *ALG1*, and *PCDH19* mutations cause the phenotypic spectrum of EIMFS to expand the genotypic spectrum. The genes *KCNT1*, *SCN2A*, *SCN1A*, *ALG1*, *ATP7A* and *WWOX* may be associated with poor prognosis. The patients presenting with MRI abnormalities, hypsarrhythmia and burst suppression in EEG may be associated with poor prognosis.

Epilepsy of infancy with migrating focal seizures (EIMFS) syndrome, which was first reported in 1995^1^, is a rare developmental and epileptic Encephalopathy (DEEs) with poor prognosis, presenting with focal seizures in the first year of life. The main clinical features are seizure onset in the first 6 months of life, occurrence of almost continuous migrating polymorphous focal seizures, combined with multifocal ictal electroencephalography (EEG) discharges, and progressive deterioration of psychomotor development^2^.

Previously, EIMFS was reported as an epilepsy syndrome with unknown etiology and poor prognosis^3^. Currently, in pace with the continuous development of genetic techniques, an increasing number of novel pathogenic genes have been shown to be related to EIMFS, including *GABRA1*, *GABRB1*, *GABRB3*, *KCNQ2*, *BRAT1*, *HCN1*, *SCN8A*, *SMC1A*, *TBC1D24*, *QARS*, *ATP1A3*, *CDKL5*, *PIGA*, *ITPA*, *AIMP1*, *KARS*, *WWOX*, *KCNT1*, *TBC1D24*, *SLC25A22*, *SCN1A*, *SCN2A*, *SLC12A5*, *PLCB1* and *PNPO*^2,4–6^. Correspondingly, precision medicine treatments were adopted and partly improved patient outcomes. For example, GoF *SCN2A* variants present with EIMFS might respond to sodium channel blockers, while LoF *SCN2A* variants present West syndrome, myoclonic atonic epilepsy, and unclassified DEEs should avoid sodium channel blockers^7^. Early quinidine intervention associated with heart monitoring and control of blood levels is among the critical factors for therapy effectiveness in EIMFS patients with *KCNT1* gain-of-function mutations^8^. Hence, this study included 36 patients who were diagnosed with EIMFS; the clinical features, etiology, treatment strategies and outcomes of these patients were determined to explore new genetic etiology and new precision medicine treatment strategies.

## Results

### Clinical phenotypic spectrum

A total of 36 patients were enrolled in this study, and all patients fulfilled the inclusion criteria. We presented the phenotypic summary of the cohort (Table 1). Among them, 25 (69.4%) were male, and 11 (30.6%) were female. Eleven patients (30.6%) had seizure onset at 4 hours to 3 days after birth. Ten (27.8%) patients had a family history of epilepsy, febrile seizure or mental retardation, or stillbirth. There were 10 (27.8%) patients with congenital heart disease, 8 (22.2%) patients with perinatal history, 1 (2.8%) patients with intracranial hemorrhage, 1 (2.8%) patients with inguinal hernia, 3 (8.3%) patient with intracranial infection, 1 (2.8%) patient with Menkes disease and 1 (2.8%) patient with congenital disorder of glycosylation, type Ik.

**Table 1:** Clinical features of the 36 children with MMPSI.

Feature	Incidence
Males: females	25:11 (2.3)
Age 4 h to 3 days at onset	11/36 (30.6%)
Age 6 months at onset	31/36 (86.1%)
**Family history of epilepsy, febrile seizure or mental retardation, stillbirth**	
Combined diseases	10/36 (27.8%)
Congenital disorder of glycosylation, type Ik	1/36 (2.8%)
Menkes disease	1/36 (2.8%)
Intracranial infection	3/36 (8.3%)
Inguinal hernia	1/36 (2.8%)
Intracranial hemorrhage	1/36 (2.8%)
Premature	1/36 (2.8%)
Congenital heart disease (open foramen ovale, patent ductus arteriosus)	10/36 (27.8%)
Perinatal history (pneumonia, HIE, intrauterine distress)	8/36 (22.2%)
Other (visual disturbance, pachygyria)	2/36 (5.6%)
**Seizure type**	
Migrating focal	36/36 (100.0%)
Tonic	21/36 (58.3%)
Spasms	8/36 (22.2%)
Clonic	5/36 (13.9%)
Others (tonic–clonic, autonomic nerve, etc.)	8/36 (22.2%)
**EEG findings**	
Seizure migration	36/36 (100.0%)
Hypsarrhythmia	13/36 (36.1%)
Burst suppression	7/36 (19.4%)
**Neuroimaging abnormal**	
Cerebral atrophy	3/36 (8.3%)
Forehead dysplasia/Widened brain space	4/36 (11.1%)
Hippocampal sclerosis Encephalomalacia foci	3/36 (8.3%) 3/36 (8.3%)
Subependymal cyst	5 /36 (13.9%)
Dysplasia of the corpus callosum	1/36 (2.8%)
Pachygyria	1/36 (2.8%)
**Genetic tests**	
Chromosome karyotype analysis	1/36 (2.8%)
Copy number variant analysis	1/36 (2.8%)
Mitochondrial genome sequencing	3/36 (8.3%)
Epilepsy genes panel	11/36 (30.6%)
Whole exome sequencing	15/36 (41.7%)
No genetic tests	10/36 (27.8%)
**Seizure control**	
Seizure free	14/36 (38.9%)
Reduced > 50%	9/36 (25.0%)
Ineffective	13/36 (36.1%)
**Outcomes**	
Severe retardation	14/36 (38.9%)
Mild-moderate retardation	13/36 (36.1%)
Normal	3/36 (8.3%)
Death	6/36 (16.7%)

HIE, Hypoxic-ischemic encephalopathy; MMPSI, Malignant migrating partial seizures of infancy.

Seizure type at onset included migrating focal seizure (36/36, 100%); tonic seizure (21/36, 58.3%); spasms (8/36, 22.2%); clonic seizure (5/36, 13.9%) and tonic-clonic, autonomic seizures and others (8/36, 22.2%). Hypsarrhythmia (classic or modified) was seen in 13 (13/36, 36.1%) patients, and a burst-suppression pattern was observed in 7 (7/36, 19.4%) patients. All patients underwent brain MRI and/or CT examinations, 17 of whom had abnormal brain imaging findings. Diffuse cerebral atrophy (with an increase in extra axial fluid) was observed in 3 children. Four cases demonstrated forehead dysplasia or widened brain space, 3 presented with hippocampal sclerosis, 3 presented with encephalomalacia foci, 1 presented with dysplasia of the corpus callosum, 5 presented with subependymal cyst, 1 presented with pachygyria and 1 presented with meningeal reinforcement. Specifically, one of the 3 children with cerebral atrophy was accompanied with hippocampal sclerosis. One child had encephalomalacia foci, hippocampal sclerosis, subependymal cyst and widened brain space.

The etiological composition of the 36 patients in this cohort was determined by the International League Against Epilepsy (ILAE) 2017 etiological classification criteria. Accordingly, there were 17 (17/36, 47.2%) patients with genetic etiology, of which 6 (6/17, 35.3%) had severe retardation, 3 (3/17, 17.6%) died and 2 (2/17, 11.8%) was normal. There were 2 (2/36, 5.6%) patients with structural etiology, both of them had severe retardation. Three patient (3/36, 8.3%) had infectious etiology, and 1 died. There were two (2/36, 5.6%) patients with metabolic etiology (both cases were inherited), of which 1 had severe retardation and 1 died. There was no immune-mediated etiology and 14 (14/36, 38.9%) cases for unknown etiology, of which 7 (7/14, 50.0%) had severe retardation and 2 (2/14, 14.3%) were normal.

### Genotypic spectrum

There were 26 (26/36, 72.2%) patients who underwent genetic testing, including 15 (15/26, 57.7%) with whole exome sequencing (WES), 11 (11/26, 42.3%) with an epilepsy gene panel, 3 (3/26, 11.5%) with mitochondrial genome sequencing, 1 (1/26, 3.8%) with chromosome karyotype analysis and 1 (1/26, 3.8%) with copy number variant (CNV) analysis. Specifically, one of the 2 patients with mitochondrial genome sequencing and WES, one of the 1 patient with mitochondrial genome sequencing, WES, chromosome karyotype analysis and CNV analysis. Seventeen (17/36, 47.2%) cases had causative variants across 11 genes (Table 2), including 6 novel EIMFS genes: de novol *PCDH19*; paternal *ALDH7A1*, *DOCK6*, *PRRT2* and *ALG1*; maternal *ALDH7A1*, *ATP7A*, *DOCK6* and *ALG1*. Five genes have been reported in previous literature, including de novo *KCNT1*, *SCN2A*, *SCN1A*, paternal *PNPO*, *WWOX*, maternal *PNPO* and *WWOX*. The most frequently implicated genes were *KCNT1* (3/36, 8.3%) and *SCN2A* (6/36, 16.7%).

**Table 2:** Variants in novel MMPSI genes and in silico analysis of pathogenicity.

Patient	Gene	Inheritance	gDNA(GRCh37/hg19)	Amino acid	rs ID	ExAc and gnomAD	Poly-phen2	SIFT	How identified	ACMG Classification
1	*KCNT1*	De novo	Chr9:138651532G > A	p.G288S	rs587777264	0;0	PD	T	WES	P
2	*KCNT1*	De novo	Chr9:138660694G > A	p.R474H	rs397515404	0;0	PD	D	WES	P
3	*KCNT1*	De novo	Chr9:138670635C > T	p.A899V	–	0;0	PD	D	panel	VUS
4	*SCN2A*	Maternal	Chr2:166246069G > A	p.R1918H	rs201718767	0.00004957203;0.000129241	B	T	WES	VUS
5	*SCN2A*	De novo	Chr2:166237654G > A	p.A1500T	–	0;0	PD	D	WES	P
6	*SCN2A*	De novo	Chr2:166237657A > G	p.M1501V	–	0;0	PD	D	panel	VUS
7	*ALDH7A1#*	Paternal Maternal	Chr5:125889987G > T Chr5:125918644dupA	p.G398W p.I139fs	rs1347421419 –	0;0 0;0	PD NA	D NA	WES	LP LP
8	*PNPO*	Paternal Maternal	Chr17:46022062G > A Chr17:46023290C > T	p.S115N p.R161C	– rs146027425	0;0 0;0.00002473207	PD PD	D D	panel	VUS VUS
9	*SCN1A*	De novo	Chr2:166909430T > C	p.L209P	NA	0;0	PD	D	panel	LP
10	*WWOX* *WWOX* *ATP7A#*	Maternal Paternal Maternal	Chr16:78133677T > C Chr16:78148874C > T ChrX:77271345DelG	p.M1? p.H78Y p.G865Dfs*5	rs758588684 – –	0;0.0000323039 0;0 0;0	PD PD PD	D D D	WES	LP VUS P
11	*DOCK6#* *DOCK6#*	Maternal Paternal	Chr19:11324989T > C Chr19:11354308G > A	p.Q1434X p.A395T	rs1194206302 rs868514448	0;0 0;0.00000822145	NA PD	NA D	WES	LP VUS
12	*PRRT2#*	Paternal	Chr16:29825016DupC	p.R217Pfs*8	rs772994486	0;0	NA	NA	WES	LP
13	*PCDH19#*	De novo	ChrX:99662806G > C	p.D264H	–	0;0	PD	D	panel	LP
14	*ALG1*	Maternal Paternal	Chr16:5123195C > A Chr16:5129065A > G	p.Q110K NA	rs774489344 rs768733117	0;0 0.000017;0	PD NA	D NA	WES	VUS LP
15	*SCN2A*	De novo	Chr2:166164381	p.C137Y	–	0;0	PD	D	WES	LP
16	*SCN2A*	De novo	Chr2:166170520	p.G429L	rs1553568987	–	PD	D	WES	LP
17	*SCN2A*	De novo	Chr2:166165304	p.A202V	–	–	PD	D	WES	LP

ACMG, American College of Medical Genetics; B, Benign; CMA, clinical microarray; CNV, Copy Number Variant; D, Deleterious; ExAC, Exome Aggregation Consortium; gnomAD, Genome Aggregation Database; LP, likely pathogenic; MMPSI, Malignant migrating partial seizures of infancy; NA, Not applicable: when the gene mutation is nonsense or frameshift mutation, the software can not predict the pathogenicity of the mutation; P, pathogenic; PD, Possibly Damaging; SIFT, Sorting Intolerant from Tolerant; T, Tolerated; VUS, variant of unknown significance; WES, Whole Exome Sequencing; #: novel gene.

### EIMFS phenotype-genotype correlation

Phenotypic data are summarized according to each gene in Table 3. We highlight the phenotypes of the five most frequently implicated genes for EIMFS here.

**Table 3:** Phenotype-genotype data of 36 patients with MMPSI.

	n	Age (M)	Age seizure onset (M)	Age at death (M)	Seizure types (n)	MRI findings: Normal (n) Abnormal (n)	EEG fingdings: Hypsarrhythmia (n); Burst suppression (n)	Effective drugs (n)	Seizure control: Seizure free (n) Reduced > 50% (n) Ineffective (n)	Outcomes: Severe retardation (n); Mild-moderate retardation (n); Normal (n); Death (n)
Cohort	36	1 year 8 month	2 month	8 month	F(36); T(20); C(5); S(8); O(8)	19; 17	13; 6	9 (OXC, VGB, CBZ, Vitamin B6, ACTH, LEV, VPA, CLP, TPM)	14; 9; 13	14; 13; 3; 6
**Patients without genetic diagnosis**										
*Gene negative	19	1 year 8 month	2 month	6 month	F(19); T(12); C3); S(1); O(3)	10; 9	6; 2	3(LEV, TPM, VPA)	6; 6; 7	8; 7; 1; 3
**Patients with genetic diagnosis**										
Gene positive	17	1 year 4 month	2 month	2 year	F(17); T(8); C(2); S(7); O(5)	9; 8	7; 4	9 (OXC, VGB, CBZ, Vitamin B6, ACTH, VPA, CLP, TPM, LEV)	9; 2; 6	6; 6; 2; 3
**Dominant Genes (11/36, 30.6%, 3 female)**										
*KCNT1*	3	1 year 4 month	1 month	2 year	F(3); T(1); S(2); O(2)	3; 0	2; 1	–	0; 0; 3	2; 0; 0; 1
*SCN2A*	6	1 year 5 month	2 days	–	F(6); T(4); S(2); O(1)	2; 4	3; 2	4(OXC, CBZ, VGB, LEV )	4; 1; 1	3; 3; 0; 0
*SCN1A*	1	6 month	2 month	6 month	F(1); T(1); S(1); O(1)	1; 0	0; 0	–	1; 0; 0	0; 0; 0; 1
*PRRT2*	1	10.5 month	3 month	–	F(1)	1; 0	0; 0	1(OXC)	1; 0; 0	0; 0; 1; 0
**Recessive Genes (5/36, 13.9%, 1 female)**										
*ALDH7A1*	1	3 year 10 month	1 month	–	F(1) C(1)	1; 0	0; 0	1(Vitamin B6)	1; 0; 0	0; 1; 0;0
*PNPO#*	1	4 year 4 month	2 days	–	F(1)	0; 1	0; 0	1(Vitamin B6)	1; 0; 0	0; 0; 1; 0
WWOX**	1	6 month	3.5 month	–	F(1)	0; 1	0; 0	1(OXC)	0; 0; 1	1; 0; 0; 0
*DOCK6*	1	2 year 7 month	6 month	–	F(1) S(1)	0; 1	1; 0	2(ACTH, VGB)	1; 0; 0	1; 0; 0; 0
*ALG1*	1	1 year	4 month	1 year	F(1) C(1)	0; 1	0; 0	–	0; 0; 1	0; 0; 0; 1
**X-linked Genes (2/36, 5.6%, 1 female)**										
ATP7A**	1	6 month	3.5 month	–	F(1)	0; 1	0; 0	1(OXC)	0; 0; 1	1; 0; 0; 0
*PCDH19#*	1	2 year 11 month	9 month	–	F(1); T(1); S(1)	1; 0	1; 0	3(TPM, VPA, CLB)	0; 1; 0	0; 1; 0; 0

ACTH, Adrenocorticotropic Hormone; C, clonic; CLP, Clonazepam; CBZ, Carbamazepine; F, focal; m, months; M, median; MMPSI, Malignant migrating partial seizures of infancyn, number; LEV, Levetiracetam; O, other (includes tonic–clonic, spasms and others); OXC, Oxcarbazepine; S, spasms; T, tonic; TPM, Topiramate; VGB, Vigabatrin; VPA, Valproic Acid; y, year; *: Gene negative patients include those who have had a genetic test and the result was negative and those who have not been tested; **: one patient has two genetic mutations.; #: The mutant was female.

#### *KCNT1* EIMFS patients (n = 3)

Three patients (3 males) with *KCNT1* variants aged 1 year and 4 months to 2 years were studied (median 1 year 4months). The median seizure onset was 1 months (range 15 days to 4 months). All had focal seizures, 1 had tonic seizures, 2 had spasms seizures, and 2 had autonomic seizures. All had normal brain imaging findings. EEG showed seizure migration in all patients, hypsarrhythmia in 2/3, and burst suppression in 1/3. Two patients had severe developmental retardation, and one died. The age of death was 2 years.

#### *SCN2A* EIMFS (n=6)

There were 6 patients with *SCN2A* EIMFS studied at 1 month to 6 years of age (median 1 years 5 months) with median seizure onset at 2 days (range 1 day to 9 months). All had focal seizures, 4 had tonic seizures,2 had spasms seizures, and 1 had an autonomic seizure. A total of 2/6 had normal brain imaging findings, and 4/6 had abnormal imaging findings. EEG data showed seizure migration in all patients, hypsarrhythmia in 3/6, and burst suppression in 2/6. The effective drugs for 6 patients with seizure control were oxcarbazepine, carbamazepine, vigabatrin and levetiracetam. Three patients had severe developmental retardation, three patients had mild-moderate development retardation, and no patients died.

#### *PNPO* and *ALDH7A1* EIMFS (n=2)

One patient (female) with *PNPO* variants was studied at 4 years and 4 months of age. Seizure onset was 2 days after birth. She had focal seizures and abnormal brain imaging findings. One patient (male) with *ALDH7A1* variants was studied at 3 years and 10 months of age. Seizure onset was 1 month. He had focal and clonic seizures and normal brain imaging. There was no hypsarrhythmia or burst suppression in the EEG of these two patients. Vitamin B6 was effective in both patients. One patient had only mild development retardation, and one patient had normal development.

#### *PRRT2* EIMFS (n=1)

**Figure 1:**
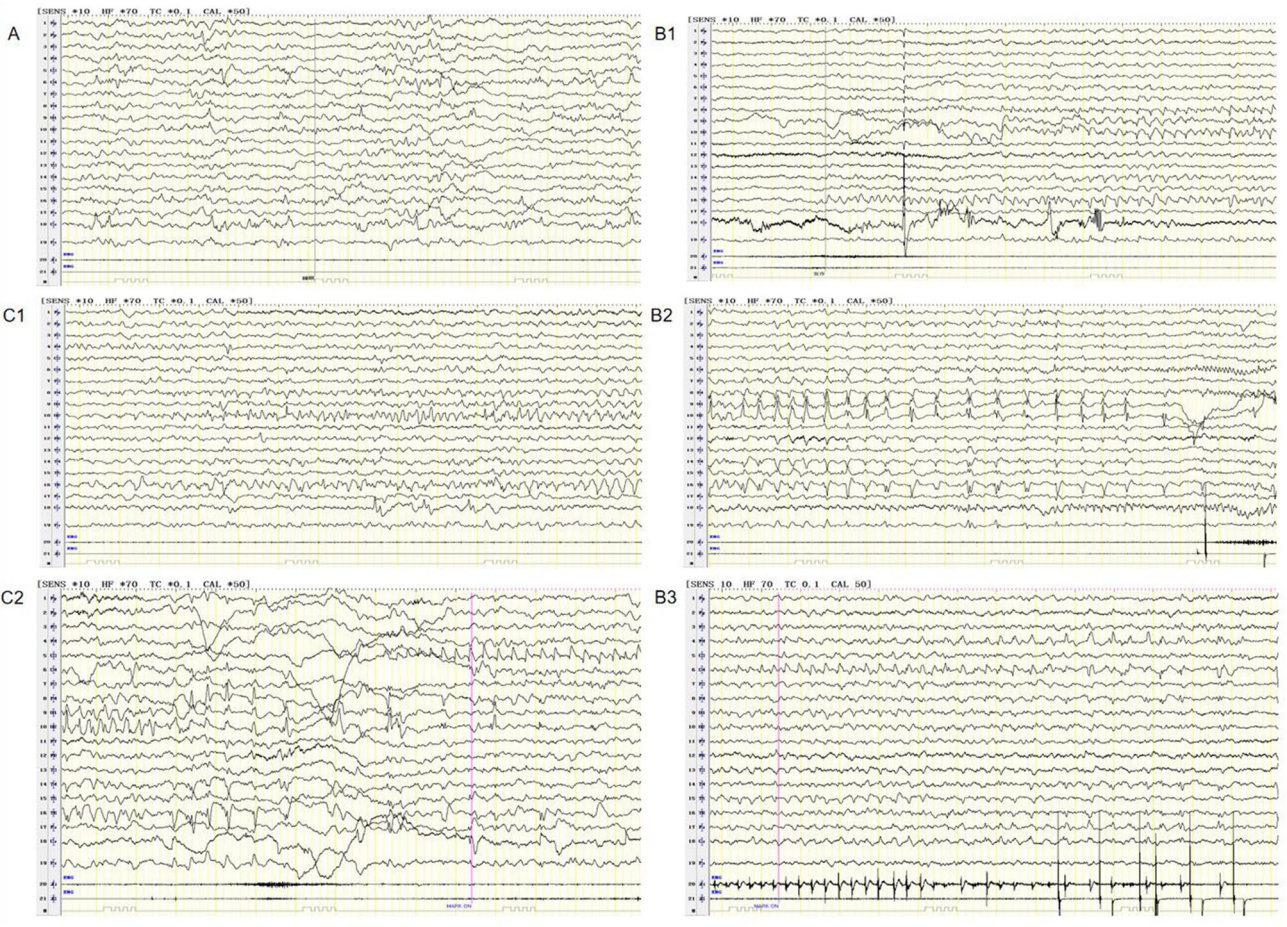
Migrating focal seizures was evident in the EEG data of the patient with *PRRT2* gene mutation. (**A**) Interictal period showed multifocal discharge; (**B1**) Focal seizures originating in the right occipital and posterior temporal regions; (**B2**) More than 10 s later, the epileptiform electrical seizure in the right posterior occipital temporal region relieved, and electrical rhythm changes appeared in the right central region. (**B3**) Convulsions of the left upper limb, simultaneous electrical seizures in the right central region; (**C1**) Focal seizures originating in the right occipital and posterior temporal regions; (**C2**) Focal seizures migrate to the left central region.

One patient (female) with *PRRT2* variants was studied at 10.5 months of age. Seizure onset was 3 months. She had migarating focal seizures and normal brain imaging findings. Migarating focal seizures and no hypsarrhythmia or burst suppression was evident in the EEG data of the patient (Figure 1). Oxcarbazepine was effective in controlling seizures in this patient, who had normal development.

### Treatment

Anti seizure medication effects on seizures were analyzed in 36 patients with EIMFS (Table 3). In addition to classical anti seizure medication, vitamin B6 and adrenocorticotropic hormones (ACTH) were used to treat EIMFS patients. Vitamin B6 allowed patients with *ALDH7A1* and *PNPO* mutations to achieve seizure-free status, oxcarbazepine was effective for patients with *SCN2A*, *ATP7A*, *WWOX*, and *PRRT2* mutations, and ACTH was partly effective for *DOCK6* mutation patients with spasms and hypsarrhythmia.

### Outcome

We obtained data regarding seizure control and motor development for 36 patients: 14/36 (38.9%) patients were seizure free, and 9/36 (25.0%) patients had seizures reduced > 50%. However, 13/36 (36.1%) patients had ineffective seizure control. 14/36 (38.9%) patients had severe retardation, 13/36 (36.1%) patients had mild-moderate retardation, and 3/36 (8.3%) patients had normal development (Table 3). Our cohort had a high mortality rate: 6/36 (16.7%) patients died at a median age of 8 months (range 3 months to 2 years). In 3/6 patients who died, pathogenic variants were identified in the following genes: *KCNT1* (1), *SCN1A* (1) and *ALG1* (1), with 3 cases remaining unsolved. The genes for ineffective seizure control and severe retardation include *KCNT1* (2), *SCN2A* (1), *ATP7A* (1) and *WWOX* (1). Genes associated with seizure-free, mild-moderate retardation or normal of mental and motor development included *PRRT2* (1), *SCN2A* (3), *ALDH7A1* (1) and *PNPO* (1).

Of the 36 patients with EIMFS, 19 had normal MRI findings, of which 9 (9/19, 47.4%) were seizure free, 9 (9/19, 47.4%) had ineffective seizure control, 7 (7/19, 36.8%) had severe retardation and 2 (2/19, 10.5%) died. Also, 17 had abnormal MRI, of which 5 (5/17, 29.4%) were seizure free, 8 (8/17, 47.1%) had ineffective seizure control, 7 (7/17, 41.2%) had severe retardation and 4 (4/17, 23.5%) died. The details are shown in Table 4.

**Table 4:** The table of MRI and prognosis.

MRI	Seizure control: Seizure free (%); Reduced > 50% (%); Ineffective (%)	Outcomes: Normal (%); Mild-moderate retardation (%); Severe retardation (%); Death (%)
Normal	9/19 (47.4%) 1/19 (5.3%) 9/19 (47.4%)	2/19 (10.5%) 8/19 (42.1%) 7/19 (36.8%) 2/19 (10.5%)
Abnormal	5/17 (29.4%) 4/17 (23.5%) 8/17 (47.1%)	1/17 (5.9%) 5/17 (29.4%) 7/17 (41.2%) 4/17 (23.5%)

MRI, Magnetic resonance imaging.

Of the 36 patients with EIMFS, 13 had hypsarrhythmia, of which 5 (5/13, 38.5%) were seizure free, 6 (6/13, 46.1%) had ineffective seizure control, 6 (6/13, 46.1%) had severe retardation, 2 (2/13, 15.4%) died and 0 (0/13, 0%) were normal; also, 7 had burst suppression, of which 1 (1/7, 14.3%) were seizure free, 1 (1/7, 14.3%) had ineffective seizure control, 3 (3/7, 42.9%) had severe retardation, 3 (3/7, 42.9%) died and 0 (0/3, 0%) were normal. 3 patients had hypsarrhythmia and burst suppression, of which 1 (1/3, 33.3%) were seizure free, 1 (1/3, 33.3%) had ineffective seizure control, 2 (2/3, 66.7%) had severe retardation, 0 (0/3, 0%) died and 0 (0/3, 0%) were normal, and 19 had no hypsarrhythmia or burst suppression, of which 8 (8/19, 42.1%) were seizure free, 7 (7/19, 36.8%) had ineffective seizure control, 5 (5/19, 26.3%) had severe retardation, 2 (2/19, 10.5%) died and 3 (3/19, 15.8%) were normal. The details are shown in Table 5.

**Table 5:** The table of EEG and prognosis.

EEG	Seizure control: Seizure free (%); Reduced > 50% (%); Ineffective (%)	Outcomes: Normal (%); Mild-moderate retardation (%); Severe retardation (%); Death (%)
Hypsarrhythmia	5/13 (38.5%); 2/13 (15.4%); 6/13 (46.1%)	0/13 (0); 5/13 (38.5%); 6/13 (46.1%); 2/13 (15.4%)
Burst suppression	1/7 (14.3%); 5/7 (71.4%); 1/7 (14.3%)	0/7 (0); 1/7 (14.3%); 3/7 (42.9%); 3/7 (42.9%)
Hypsarrhythmia and burst suppression	1/3 (33.3%); 1/3 (33.3%); 1/3 (33.3%)	0/3 (0); 1/3 (33.3%); 2/3 (66.7%); 0/3 (0)
No hypsarrhythmia and burst suppression	8/19 (42.1%); 4/19 (21.1%); 7/19 (36.8%)	3/19 (15.8%); 9/19 (47.4%); 5/19 (26.3%); 2/19 (10.5%)

EEG, Electroencephalogram.

## Discussion

EIMFS is characterized by nearly continuous seizures involving multiple independent areas of both hemispheres with arrested psychomotor development. It is an age-dependent, often overlooked syndrome among the epileptic encephalopathies that can occur within the first 6 months of life^9^. EIMFS has clinical phenotypic overlap with other epileptic encephalopathies in early infancy, such as early infantile epileptic encephalopathy, early myoclonic encephalopathy, and infantile spasms^7,10^. EIMFS has no specific neuroimaging changes reported in the current literature. We described the phenotype spectrum of 36 patients with EIMFS in this study. All patients had clinical seizure migration associated with a significant impact on development. We identified several key phenotypic features that have only been rarely reported in the EIMFS phenotypic spectrum: 11 (11/36, 30.6%) patients had suspected in utero seizures with postnatal seizures described between 4 hours and 3 days, 8 (8/36, 22.2%) patients had epileptic spasms, 7 (7/36, 19.4%) had a burst-suppression pattern in EEG activity, 1 patient had congenital disorder of glycosylation—type Ik, 1 patient had Menkes disease. Furthermore, it is mostly believed that the etiology of EIMFS is caused by genetic mutations^9^. According to our etiological analysis, the genetic etiology accounted for 17/36 cases, and structural etiology accounted for 2/36 cases. Analysis of etiology and prognosis results suggests that structural causes may be related to poor prognosis. Our data expand the clinical phenotype of EIMFS.

We describe the genotypic spectrum of 17 patients with EIMFS. We highlight the extensive genetic heterogeneity of EIMFS, which is similar to that in other epilepsy syndromes, such as infantile spasms (West syndrome) and Lennox-Gastaut syndrome, but with a considerably higher yield on current testing. We identified the etiology in 47.2% of our cohort, including 6 novel EIMFS genes. The most commonly involved genes were *KCNT1* (8.3%) and *SCN2A* (16.7%), together explaining 25.0% of EIMFS cases. These studies highlight the importance of classifying a patient's epilepsy syndrome, which influences genetic testing and interpretation^11^. Epileptic spasms have only been rarely reported in EIMFS patients^2^. In this series, 8/36 patients (22.2%) had epileptic spasms, including those with *KCNT1*, *SCN2A*, *SCN1A*, *DOCK6* and *PCDH19* variants.

We discovered 6 novel EIMFS genes in our cohort - de novo *PCDH19*; paternal *ALDH7A1*, *DOCK6*, *PRRT2* and *ALG1*; and maternal *ALDH7A1*, *ATP7A*, *DOCK6* and *ALG1 -* encoding a wide range of proteins*.* These genes have not been described in patients with EIMFS, but they have been associated with other neurological diseases. Considering that one of our patients was a female and had a de novo mutation of the *PCDH19* gene, which was consistent with the X-linked genetic classification and the common clinical phenotype of spasms seizure and delayed motor development^12^, the software predicted the likely pathogenicity of the mutation; thus, these findings suggested that the *PCDH19* gene could be associated with EIMFS. *ALDH7A1* gene is associated with autosomal recessive pyridoxine-dependent epilepsy^13^. Our patient's clinical phenotype was recurrent epileptic seizures, accompanied by developmental delay, and vitamin B6 treatment was effective. The inheritance pattern was consistent with family co-segregation, and the software predicted it as likely pathogenic. Therefore, it can be considered as the causative gene of EIMFS. In future studies, it is hoped that more case reports, or functional studies, can confirm the pathogenicity of this variant. *DOCK6* gene is associated with autosomal recessive Adams-Oliver syndrome-2, which is characterized by aplasia cutis congenita (ACC) as well as terminal transverse limb defects (TTLD) in addition to variable involvement of the brain, eyes, and cardiovascular system^14^. Our patient had developmental delays and recurrent seizures, which may be related to brain development disorders. Therefore, we could not determine whether the *DOCK6* gene was the causative gene of EIMFS, and further data are needed for verification. It has been previously thought that *PRRT2*-associated paroxysmal movement disorders (*PRRT2*-PxMD) are mostly manifested as infantile-onset epilepsy and post-adolescent movement disorders, and most of them have a good prognosis and do not affect cognitive development. In addition, *PRRT2* pathogenic variants have been identified in other childhood-onset movement disorders and different types of seizures, suggesting that the understanding of the spectrum of *PRRT2*-PxMD is still evolving^15^. The patient with this mutation in our cohort was very interesting. He presented with very frequent cluster seizures when he was 3 months old, video-EEG demonstrated migrating focal seizures and typical background characteristics of EIMFS, and he experienced obvious cognitive retardation after the onset of epilepsy. He achieved seizure-free status after the administration of oxcarbazepine, and follow-up showed normal cognitive development. The patient had a paternal mutation of the *PRRT2* gene—his father had symptoms of dystonia in his youth—and the software predicted the likely pathogenicity of the mutation. We considered that the *PRRT2* gene was the causative gene of EIMFS, suggesting that *PRRT-2* spectrum diseases could also manifest as a very severe epileptic encephalopathy phenotype, but after effective treatment, the disease process could be quickly reversed and the prognosis was good. In the 10th patient, two genetic variants were detected simultaneously, namely, *WWOX* and *ATP7A*. The clinical phenotypes of *WWOX* gene reported in the literature were autosomal recessive epileptic encephalopathy, early infantile 28, esophageal squamous cell carcinoma, somatic and spinocerebellar ataxia, and autosomal recessive 12^16,17^. The *WWOX* gene was EIMFS-related genes reported in previous literature, which theoretically can cause epileptic encephalopathy. The clinical phenotypes of *ATP7A* gene reported in the literature were X-linked Menkes disease, occipital horn syndrome, distal spinal muscular atrophy 3^18–20^. This patient also had a significant decrease in blue copper protein, had hair and skin color changes and could have been diagnosed with Menkes disease. At present, the epileptic seizure forms of Menke's disease reported in the literature include focal clonic seizures, myoclonic, and tonic seizures, infantile spasms, and no migrating focal seizures have been reported^21^. We could not confirm whether it was a copathogenic gene. *ALG1* gene is associated with autosomal recessive congenital disorder of glycosylation type Ik^22,23^, which manifested as feeding problems and diarrhea, muscular hypertonia, refractory seizures, recurrent episodes of apnea, cardiac and hepatic involvement and coagulation anomalies^24^. The clinical characteristics of this patient were consistent with the above core symptoms, the genetic results were consistent with the law of autosomal recessive inheritance, and the software predicted the likely pathogenicity of the mutation. We considered the *ALG1* gene to be a newly pathogenic gene of EIMFS.

Most patients with EIMFS are refractory to anti-seizure medication, but some have shown good progression or a near satisfactory response to treatment. The anti-seizure medication used alone or in combination with one another that may achieve seizure control or reduction are potassium bromide, levetiracetam, ACTH, stiripentol, clonazepam, and rufinamide^25^. Mikati et al. reported that quinidine is effective in the treatment of patients with *KCNT1* gene mutations in EIMFS^26^. Some studies have found that a correlation between age at disease onset, response to sodium channel blockers and the functional properties of mutations in children with *SCN2A*-related epilepsy. The use of sodium channel blockers was often associated with clinically relevant seizure reduction or seizure freedom in children with early infantile epilepsies (<3 months), whereas other antiepileptic drugs were less effective. In contrast, sodium channel blockers were rarely effective in epilepsies with later onset (≥3 months) and sometimes induced seizure worsening^27^. At present, there are few literature reports on the use of corresponding effective drug treatments for patients with different EIMFS gene mutations. In our cohort, we determined that vitamin B6 could allow patients with *ALDH7A1* and *PNPO* mutations to achieve seizure-free status. Oxcarbazepine was effective for patients with *SCN2A*, *ATP7A*+*WWOX*, and *PRRT2* mutations. Among our 6 children with *SCN2A* gene mutations, 4 of them were onset before 3 months of age, and the seizure control effect was good, which was consistent with the data reported in the literature. ACTH was partly effective for patients with *DOCK6* mutations who had spasms and hypsarrhythmia.

While seizure outcomes and developmental prognoses are generally poor in EIMFS, there are rare reports of mildly affected patients^28^. In our study cohort, the incidence of poor prognosis was also relatively high; 6/36 (16.7%) patients died, and the related pathogenic genes were *KCNT1*, *SCN1A*, *ALG1*. 14/36 (38.9%) patients had severe retardation, and the genes for ineffective seizure control and severe retardation included *KCNT1*, *SCN2A*, *WWOX* and *ATP7A*. The results indicated that the related pathogenic genes *KCNT1*, *SCN1A*, *ALG1*, *SCN2A*, *WWOX* and *ATP7A* may be associated with ineffective seizure control and poor prognoses. While all patients experienced refractory epilepsy early in the course of the disease, 3/36 (8.3%) patients had normal mental and motor development. Genes associated with seizure-free, mild-moderate retardation or normal of mental and motor development included *PRRT2*, *SCN2A*, *ALDH7A1*, *PCDH19* and *PNPO*.

In addition, we compared the association of MRI abnormalities, hypsarrhythmia and burst suppression in EEG with poor prognosis. The results found that patients with EIMFS characterized by abnormal MRI, hypsarrhythmia and burst suppression in EEG have a higher incidence of ineffective seizure control, severe retardation and a higher mortality rate. The results suggest that EIMFS patients who present with abnormal MRI, hypsarrhythmia and burst suppression in EEG may be associated with ineffective seizure control and poor prognosis. We need to further expand the sample to analyze and confirm these correlations.

## Conclusions

In conclusion, our study expands the EIMFS clinical phenotype and genotype spectra. EIMFS patients who present with abnormal MRI findings, hypsarrhythmia and burst suppression in EEG may be associated with ineffective seizure control and poor prognosis. Etiological analysis showed that in addition to genetic mutations, structural etiology may be associated with poor prognosis. This study is the first to report that *ALDH7A1*, *ATP7A*, *DOCK6*, *PRRT2*, *ALG1*, and *PCDH19* mutations cause the phenotypic spectrum of EIMFS. The genes *KCNT1*, *SCN1A*, *ALG1*, *SCN2A*, *WWOX* and *ATP7A* may be associated with ineffective seizure control and poor prognosis. Through early diagnosis with genetic tests and the administration of the corresponding precise treatment, the outcomes of EIMFS can be notably improved.

## Methods

### Participants and phenotyping

Our EIMFS cohort comprised 36 patients from Hunan Children's Hospital and Qilu Hospital of Shandong University. A multicenter retrospective case study was performed over a 10-year period (January 2010 to January 2020). The parents of the patients provided written, informed consent. This study was approved by the Medical Ethics Committee of Hunan Children’s Hospital and Qilu Hospital of Shangdong University.

According to the clinical characteristics of EIMFS described by Coppola^1^, the inclusion criteria are as follows: (1) onset within 6 months after birth; (2) migrating focal seizures at onset; (3) multifocal seizures intractable to conventional antiepileptic drugs; (4) the EEG during the onset period is characterized by multifocal discharges, migrating in one hemisphere or between the two hemispheres, involving multiple parts, and the clinical onset is closely related to the time and location of the EEG discharge; and (5) delayed developmental progress or signs of psychomotor regression associated with seizure onset. The phenotypic information of all patients was collated on clinical presentation, disease course, EEG, neuroimaging, treatment strategies and the results of neurometabolic and diagnostic genetic investigations. All patients were followed up every 1-6 months by telephone or outpatient department.

It should be noted in particular that one of our patients met the inclusion criteria except for the onset age of 9 months. Considering that EIMFS with onset age of 9 months has been reported in literatures, we still included this patient in our study cohort.

### Genetic tests

Genetic testing was carried out using chromosome karyotype analysis, CNV analysis, mitochondrial genome sequencing, epilepsy gene panels and WES. CNV analysis was performed with the Illumina HumanOmniZhonghua-8 Bead Chip; Mitochondrial genome sequencing was subsequently performed on an Illumina HiSeq 2000 platform (Illumina, San Diego, CA, USA); details were provided by our team previously^29^. The epilepsy gene panel contained 265 epilepsy-associated genes was performed on methods previously reported by Lemke et al^30^. WES was also performed based on methods previously reported by our team^29^ by using Illumina HiSeq X Ten (Illumina, San Diego, CA, USA) with 150-bp paired-end reads.

### Ethics approval and consent to participate

The study was performed in accordance with the Declaration of Helsinki, with the approval of the study protocol by an independent ethics committee or institutional review board at each site. All patients provided written informed consent before participation.

### Consent for publication

All authors have approved the final article and its publication.

## Data Availability

The datasets used and/or analyzed during the present study are available from the corresponding author on reasonable request.

## Abbreviations

ACTH: Adrenocorticotropic hormones
ACC: Aplasia cutis congenita
BFIE: Benign familial infantile epilepsy
CNV: Copy number variant
EEG: Electroencephalography
EIEE-9: Early infantile epileptic encephalopathy-9
HM: Hemiplegic migraine
EIMFS: Epilepsy of Infancy with Migrating Focal Seizures
HIE: Hypoxic-ischemic encephalopathy
ILAE: International League Against Epilepsy
IC: Infantile convulsions
MT I: Mannosyltransferase I
PRRT2-PxMD: PRRT2-associated paroxysmal movement disorders
PKD: Paroxysmal kinesigenic dyskinesia
TTLD: Terminal transverse limb defects
VUS: Variant of unknown significance
WES: Whole exome sequencing
